# Re-visioning Ultrasound through Women’s Accounts of Pre-abortion Care in England

**DOI:** 10.1177/0891243215590101

**Published:** 2015-10

**Authors:** Siân M. Beynon-Jones

**Affiliations:** University of York, United Kingdom

**Keywords:** abortion, ultrasound, women’s experiences, pregnancy

## Abstract

Feminist scholarship has demonstrated the importance of sustained critical engagement with ultrasound visualizations of pregnant women’s bodies. In response to portrayals of these images as “objective” forms of knowledge about the fetus, it has drawn attention to the social practices through which the meanings of ultrasound are produced. This article makes a novel contribution to this project by addressing an empirical context that has been neglected in the existing feminist literature concerning ultrasound, namely, its use during pregnancies that women decide to terminate. Drawing on semi-structured interviews with women concerning their experiences of abortion in England, I explore how the meanings of having an ultrasound prior to terminating a pregnancy are discursively constructed. I argue that women’s accounts complicate dominant representations of ultrasound and that in so doing, they multiply the subject positions available to pregnant women.

For several decades, feminist scholarship has mapped and deconstructed anti-abortion claims that ultrasound images of pregnancy provide “objective” evidence of fetal personhood (see, e.g., Condit 1990; Daniels 1993; Duden 1993; Hartouni 1997; Hopkins, Zeedyk, and Raitt 2005; Petchesky 1987; Roberts 2012a; Science and Technology Subgroup 1991; Sheldon 1997; Stabile 1998; Taylor 1998, 2008). By demonstrating the social practices through which the meanings of these images are constructed, such research disrupts anti-abortion claims that ultrasound can be used “to see and know *the* truth from a single perspective” (Roberts 2012a, 133 [emphasis in original]). This article contributes to this project by exploring the discursive practices through which women in England construct the experience of having an ultrasound prior to abortion. In addition to addressing a context of pregnant embodiment that has been neglected by empirical feminist research concerning ultrasound (Gerber 2002; Kimport et al. 2012), it argues that engagement with this context offers novel opportunities to destabilize dominant representations of this sociotechnical practice.

## Deconstructing Dominant Representations of Ultrasound

Anti-abortion deployments of ultrasound imagery have been conceptualized as part of a broader reframing of abortion as an object of medical, rather than religious, knowledge (Franklin 1991; Petchesky 1987). In England, Scotland, and Wales, medical expertise is made central to the regulation of abortion through the 1967 Abortion Act, as amended by the 1990 Human Fertilisation and Embryology Act. Two doctors must agree that the procedure is necessary on the grounds of a pregnant woman’s health or that of her fetus. These grounds become more limited beyond 24 weeks’ gestation, which forms the upper time limit on most abortions. Doctors can legally interpret the clinical grounds for abortion very broadly (Sheldon 1997), and research suggests that in practice, abortion is generally conceptualized as a pregnant woman’s decision (Lee 2003). Nonetheless, feminist theorists critique the law’s construction of abortion as a deviant act that requires regulation by medical experts (Boyle 1997; Sheldon 1997). In addition to its problematic portrayal of women’s reproductive decision making, this framework renders abortion provision vulnerable to challenge on the basis of claims concerning medical knowledge and practice (Science and Technology Subgroup 1991; Sheldon 1997).

In recent decades, this vulnerability has been underscored by repeated (albeit, to date, unsuccessful) anti-abortion campaigns. One such campaign, which has focused on lowering the legal time limit on abortion, has used developments in ultrasound imaging to claim that the fetus is visibly “a person” at gestations below 24 weeks. As Palmer highlights, such arguments depend upon the “conflation of seeing with knowing” (Palmer 2009b, 174) and ignore non-visual forms of knowledge about pregnancy (i.e., a pregnant woman’s embodied knowledge of its social sustainability). They also imply that medical imaging technologies provide unmediated, objective knowledge (Palmer 2009b; Roberts 2012a). Yet, as Petchesky (1987) demonstrates, images produced using ultrasound *construct* the fetus as an autonomous individual “dangling in space, without a woman’s uterus and body and bloodstream to support it” (Petchesky 1987, 63). In other words, this technology generates a particular visualization of pregnancy from which pregnant women are erased.

Hopkins, Zeedyk, and Raitt (2005) argue that in addition to deploying this “fetal-centred” (Steinberg 1991, 179) depiction of pregnancy, anti-abortion groups also use a particular (unacknowledged) vantage point to construct the meaning of *viewing* ultrasound images. Opponents of abortion often draw on the accounts of “the willingly pregnant,” who describe ultrasound as a pivotal moment of emotional bonding during which the “personhood” of an eagerly anticipated fetus is made visible (Hopkins, Zeedyk, and Raitt 2005). Mobilizing the social construction of emotion “as ‘unmediated’ and ‘authentic’” (Hopkins, Zeedyk, and Raitt 2005, 395), anti-abortion campaigners position such responses as objective evidence of fetal personhood and argue that if women requesting abortion were shown ultrasound images of their pregnancies, they would inevitably share this emotional response and be dissuaded from the procedure.

Hopkins, Zeedyk, and Raitt (2005) offer an important critique of anti-abortion attempts to define the meaning of all encounters with ultrasound using the accounts of women who plan to carry their pregnancies to term.^1^ Arguably, however, their analysis neglects a broader problem, namely, the limiting subject position that dominant depictions of ultrasound as a tool of “maternal-fetal bonding” construct for *all* pregnant women. Taylor (1998, 2008) traces the history of this depiction, connecting it to early medical advocates of the technology who argued (on the basis of highly debatable evidence) that viewing ultrasound images would cause a woman to form an emotional “bond” with her fetus, producing better compliance with medical advice during prenatal care. Over time, this construction of ultrasound has become entrenched through prenatal care practices (Taylor 1998, 2008). Moreover, it has traveled beyond the clinic (e.g., in advertising and other forms of popular culture), to the extent that ultrasound is now understood as a joyful social rite of passage in the transition to contemporary Western motherhood (Roberts 2012a; Taylor 1998, 2008). As Roberts highlights, this narrow framing prevents the multiplicity of pregnant women’s encounters with ultrasound from being “voiced and acknowledged, including . . . unpleasurable or indifferent experiences of viewing ultrasound images” (2012a, 133).

In this article, I draw on these insights concerning the social construction of ultrasound to explore women’s interview accounts of encountering the technology prior to abortion. I illustrate that women’s accounts are often oriented to constructions of ultrasound as a moment of joyful maternal spectatorship of fetal personhood. However, I also suggest that they destabilize this dominant representation by offering alternative depictions of this sociotechnical practice.

### Ultrasound in Pregnancies that Women Plan to Carry to Term

A key way in which existing feminist research challenges dominant representations of ultrasound is by exploring the contingencies of its use in pregnancies that women plan to carry to term. For example, Mitchell (2001) illustrates that far from being an unmediated event, women’s encounters with prenatal ultrasound in Canada are produced through their interactions with sonographers. These practitioners narrate the unintelligible two-dimensional images on screen in a manner (e.g., describing the fetus as an individual with distinct personality traits) that enables women to construct an emotionally significant picture of “their baby” (Mitchell 2001).

In her analysis of prenatal ultrasound in the United States, Taylor (1998, 2008) draws attention to another contingency that is erased from anti-abortion portrayals of ultrasound as a tool of maternal-fetal bonding. She points out that women are offered the opportunity to meet and bond with their fetuses as part of the objectifying process of prenatal diagnosis, during which the fetus is subjected to “a critical scientific gaze that evaluates its condition” (Taylor 1998, 24). Because few conditions diagnosed prenatally can be treated, the implicit function of this process is to enable women to terminate their pregnancies if “abnormalities” are discovered.

Taylor’s analysis suggests that in the United States, the practice of ultrasound centers on a “prenatal paradox” (Taylor 1998, 16) whereby the fetus is simultaneously objectified and personified as a separate individual. However, cross-culturally comparative research demonstrates that ultrasound is practiced very differently in other contexts. For example, Mitchell and Georges (1997) find that in Greece, ultrasound is provided with minimal narration and is framed *primarily* as a medical technology designed to detect fetal abnormalities. Other studies have similarly shown how different understandings of the processes via which personhood is produced, as well as the meaning of medical knowledge, create locally diverging prenatal “ultrasounds” (Gammeltoft 2007; Harris et al. 2004; Ivry 2006; Morgan 2000; Saetnan 2000).

Some authors have extended this form of analysis to alternative contexts of pregnant embodiment. Layne (2003) reflects on the implications of prenatal ultrasound for women who go on to experience miscarriage, stillbirth, or neonatal death. Likewise, Rapp (2000) and Mitchell (2004) address women’s experiences of receiving a diagnosis of fetal abnormality *during* an ultrasound scan. In contrast, little is known about how experiences of ultrasound are negotiated in the context of pregnancies that women decide to terminate (Gerber 2002; Kimport et al. 2012).

### Ultrasound in Pregnancies that Women Decide to Terminate

In England, ultrasound is a routine part of pre-abortion care. The Royal College of Obstetricians and Gynaecologists’ (2011, 52) clinical guideline *The Care of Women Requesting Induced Abortion* suggests that although it is no longer necessary, the routine use of pre-abortion ultrasound to confirm the gestation of a pregnancy became entrenched following the introduction of medical abortion (a procedure originally associated with a strict gestational time limit).

The guideline recommends that women should be asked whether they wish to view their ultrasound image (Royal College of Obstetricians and Gynaecologists 2011, 53). This recommendation is made on the basis of quantitative findings from studies in South Africa (Bamigboye et al. 2002) and Canada (Wiebe and Adams 2009) demonstrating that many^2^ women want to view their ultrasound images (although many do not), and that most who do describe this as a positive experience that does not alter their decision about abortion (see also Graham, Ankrett, and Killick 2010; Kimport et al. 2013). Related findings are highlighted in a qualitative study by Kimport et al. (2012), who explored women’s emotional reactions to viewing their ultrasound image before abortion in the United States, and found that several women deliberately sought, and valued, this experience as part of the process of ending a pregnancy. As Kimport et al. (2012) note, such findings disrupt anti-abortion predictions about the impact of ultrasound on women seeking abortions.

However, while its findings challenge claims that ultrasound produces *predictable* responses in pregnant women, this small body of research is also limited in key respects. In investigating women’s “responses” to or “preferences” about ultrasound viewing, it constructs this process as an asocial encounter between an individual pregnant woman and a neutral image of her pregnancy, whose “impact” can be empirically determined. Notably, Kimport et al. do highlight the importance of understanding “how the context of . . . viewing may influence women’s interpretation of her ultrasound image” (Kimport et al. 2012, e516). Nevertheless, their emphasis is on how the *individual* circumstances of pregnancy may alter the “effect” of ultrasound images. As with the other studies of pre-abortion ultrasound outlined above, their analysis does not consider how ultrasound images, and women’s encounters with them, are constituted through available social accounts of their meaning.

In contrast, this article takes as its point of departure the insight of feminist theorizations of ultrasound outlined above, namely that “the technological is profoundly social and political” (Taylor 2008, 25). Rather than asking how women “respond” to pre-abortion ultrasound, it explores the terms in which it is possible to make sense of, and represent, the meaning of this sociotechnical practice.

## Methods

The analysis presented here is drawn from 23 semi-structured interviews that were conducted as part of a broader study that explored women’s experiences of abortion in England. The research was approved by an NHS Research Ethics Committee and the University of York’s Economics, Law, Management, Politics and Sociology Ethics Committee.

I planned to recruit participants via clinics that provide abortion, with the study information being given to women on the day of their procedure, and interviews being conducted two to six weeks afterwards. However, because recruitment in this context proved very difficult, I also advertised for participants via newspapers and social media. These combined strategies produced a sample of 28 women (17 of whom were recruited via clinics and 11 of whom were recruited via external ads). Of these participants, 23 described having an ultrasound as part of their experience of abortion; their accounts form the focus of this article. All participants were offered a gift of £20 to thank them for their time.

Interviews took place either by phone or face-to-face (depending on participants’ preferences) and were recorded and transcribed verbatim, except in one case where the recording device failed and detailed notes were written up immediately afterwards. Participants’ identities have been anonymized using interview numbers. This represents the balancing of my own (ongoing) concern about the ways that numbers disembody research participants, with the anxieties that many women expressed about the concealment of their identities. Following these interviews, I decided that although unlikely, the consequences of inadvertently identifying a participant through using pseudonyms (e.g., if a woman had concealed her full name from me) were too serious to risk. In future research, I would deal with this issue differently, by involving women in the process of anonymizing their identities (e.g., by asking them to choose a pseudonym).

Interviews drew on a topic guide that had been developed on the basis of existing literature concerning women’s experiences of abortion. I aimed to give participants the space to define the salient aspects of their experiences by beginning with a very open-ended question: “Can you tell me a bit about what happened when you first thought that you might be pregnant?” In response, women typically provided long accounts, about which I then asked follow-up questions. In most interviews, women spontaneously described “having a scan” prior to abortion. After it became clear that this represented a significant part of women’s experiences, if accounts of ultrasound were not offered spontaneously during interviews, I began to ask women if they had received a scan and, if so, what this was like.

As anticipated in the ethical review of the study, a small minority of participants became distressed when talking about their experiences. If this happened, I made sure that they wanted to continue with the interview (which all participants did). At the end of the interview, I also checked that they were aware of potential support services available to them. Significantly, those who expressed distress highlighted the importance of being *allowed* to talk about emotionally upsetting, yet socially silenced, experiences.

In order to explore whether the study included participants with varying backgrounds, a short questionnaire was used to collect basic demographic information. Using British census categories, the majority of the participants identified as “White British/Other White background” (n=21). One participant identified as “Black/Black British–Caribbean,” and one identified as having a specific “Mixed background.” Participants were generally highly educated (most had, or were undertaking, undergraduate degrees). Women’s ages at the time of their abortions ranged from approximately 17–36 years, and the gestation of their pregnancies ranged from approximately 5–22 weeks.

The time elapsed between participants’ abortions and their interviews ranged from approximately 3 weeks to 13 years. This means that those who took part in the study had very different opportunities to reflect upon their encounters with ultrasound prior to being interviewed. Additionally, it seems likely that the meaning of these experiences will have changed in relation to subsequent life events. Nonetheless, in terms of the analysis presented here, there were no discernible differences between the accounts of women for whom abortion was a relatively recent event and those for whom it had taken place many years previously.

Most women described experiences of ultrasound within the context of pre-abortion care. However, four women also described having an ultrasound during prenatal care. In three cases, this was because they had originally planned to carry their pregnancies to term, but their circumstances changed during pregnancy (for one participant, this occurred when a lethal fetal condition was diagnosed during the scan). For one woman, participation in a prenatal scan was the result of pressure from her partner to keep her pregnancy, a situation that is explored further below.

The analysis that follows draws on Potter and Wetherell’s (1987) approach to discourse analysis in the sense that it explores the “interpretative repertoires” through which women construct their encounters with ultrasound prior to abortion. An interpretative repertoire, Potter and Wetherell suggest, is “a lexicon or register of terms and metaphors drawn upon to characterise and evaluate actions and events” (1987, 138). In identifying the interpretative repertoires that women draw upon to describe pre-abortion ultrasound, my aim is to understand what it “is possible to say” (Edley 2001, 201) about this experience.

## A Technology of Situated Visual Relationships

As described above, dominant representations of ultrasound routinely position pregnant women as joyful maternal witnesses to visualizations of fetal personhood. Across many of the interviews, participants drew on elements of this depiction, in particular, the characterization of ultrasound as an emotionally significant visual encounter between a pregnant woman and her fetus. However, they also reworked and contested dominant representations using (what I have termed) an interpretative repertoire of “situated visual relationships.” Through this repertoire, they emphasized various ways in which the process of viewing ultrasound is socially “situated” (Haraway 1988). In doing so, they contested the “conflation of seeing with knowing” (Palmer 2009b, 174) and also offered representations of ultrasound as something other than an experience of maternal-fetal bonding. Below, I provide examples of participants’ use of this repertoire.

### Resisting the Script: Accounts of Deliberate Not-Looking

Many participants described the experience of having an ultrasound as one of trying to navigate and resist a rigid sociotechnical “script” (Akrich 1994) that was incompatible with their own experiences of pregnancy:

I purposely chose not to look . . . it was very much like I’m not looking. And they gave me the option. They asked, “Do you want to see?” And I was, like, “No.” The *sensation* of having the stuff put on you—the jelly—it’s almost, like, the first time of, like, this is real. Because even though you’re not seeing the screen you’re imagining what it would look like. Because you’ve seen enough programmes of that. And I think it’s probably meant to be one of the most joyous things of looking at a screen and seeing your baby move. And I don’t know until I have my first child that’s wanted if that will have taken away from it or not. (Interview 15)

I remember not wanting to look at it or anything. . . . Because it does make it—if anything that probably did upset me, that bit, because I think—especially because my boyfriend had come with me and I think he came in the room as well and I thought this is a little bit like, you know, when you sort of are happy to be having one. And I think that bit did make me a bit uncomfortable with the whole thing. You know, with the decision. So, yeah, I didn’t sort of look at the screen . . . that bit [*the ultrasound*] probably did upset me the most because it’s exactly like you see on TV when people are, you know, happy to be having a baby. You go, don’t you, the jelly on your belly and then you’re like—so, yeah, that bit probably was the bit that made me think, ooh . . . because, like, you relate that as well from growing up, seeing happiness, don’t you, as well. I think that’s linked to people being really happy and you’re going to, you know [*end the pregnancy*]. . . . It’s never really—you never see people that are obviously not happy that they are [*pregnant*]. (Interview 21[italicized text in brackets is my annotation])

On the one hand, these accounts of how ultrasound is “meant to be” reify normative depictions of the technology as a tool of maternal-fetal bonding and position women’s encounters with it in the context of abortion care as deviant. On the other, by articulating a disjuncture between expectations and experiences of ultrasound, they situate encounters with this technology in two key ways. First, in rejecting the act of looking at the viewing screen as inappropriate to their own pregnancies, these participants depict ultrasound as an affective experience that depends on social context. Joyful recognition of fetal personhood, they argue, is not intrinsic to the experience of having an ultrasound. Rather, the *pursuit* of joyful visual encounters with the fetus requires the context of a “wanted” pregnancy, in which women actively seek to construct and develop maternal-fetal relationships. Second, these participants construct experiences of ultrasound (whether “joyful” or “difficult”) as inseparable from dominant social representations of its meaning. Strikingly, although neither of the women quoted above looked at the viewing screen, both portray the experience of having a scan as a process of “seeing” normative depictions of ultrasound in popular culture. This pattern was repeated across many of the interviews, where participants talked about the significance of feeling the ultrasound gel being placed on their abdomens, the setup of the room, the presence of their partners during the scan, the movements made by the sonographer, and the fact that looking at the screen was an ever-present possibility. Such practices were presented as synecdoches for a ritual affective experience central to popular cultural portrayals of pregnancy: the excitement and joy of “looking at a screen and seeing your (wanted) baby move.” Women often described their discomfort (and, in some cases, distress) at being drawn into this ritual through these practices, while simultaneously being excluded from it because of their feelings in relation to their own pregnancies. Their accounts are suggestive of the difficulties involved in trying to reject dominant depictions of ultrasound. Indeed, Interviewee 21 (above) comments explicitly on this issue, noting how on television ultrasound is “linked to being really happy” and there are no alternative representations of this practice.

### Subverting the Script: Accounts of Deliberate (or Desired) Looking

In the accounts explored above, women reject the possibility of looking at the viewing screen as inappropriate to the context of their own pregnancies. In contrast, other participants described ultrasound as a technology that can facilitate emotionally significant visual encounters with the fetus prior to abortion. Crucially, however, by constructing what it means to encounter the fetus using a repertoire of “situated visual relationships,” they challenged the limited subject position made available by anti-abortion depictions of this process.

One participant described ultrasound as a visual encounter that her partner was able to enlist against her in his attempt to persuade her to continue with her pregnancy. Although she knew that she wanted to request an abortion, he pressured her to delay this decision until after her first prenatal ultrasound, during which he encouraged her to watch the viewing screen:

I saw the baby sucking its thumb and saw it move. And I went into the scan hoping there was no heartbeat so that told me a lot as well. Because I thought if there’s no heartbeat at least then it’s nature taking its course rather than me having to do what I did. But no, he was, like, looking in awe at the screen and I was just looking away. And then he said, “Look, because that’s what we’re here for,” so I looked. And you get that feeling of, that’s my baby, but at the same time you get that feeling of it just confirms what you already know. And I think he thought because of my maternal instinct . . . that as soon as I saw it and I saw it move and saw it on the screen and it became more real, I think he thought that that would just [flip] my mind but it didn’t. It kind of confirmed. Because if I could still think it when I saw what I saw, to me that says it was the right decision for me. (Interview 3 [non-italicized text in brackets indicates unclear word(s)])

This extract vividly portrays the troubling implications of the “democratization” (Sandelowski 1994, 242) facilitated by ultrasound. Studies of heterosexual couples’ experiences of ultrasound during prenatal care illustrate that this technology can facilitate men’s involvement in their partner’s pregnancies (Draper 2002; Reed 2012; Sandelowski 1994). However, Sandelowski (1994) suggests that such involvement may be double-edged; by facilitating men’s ability to see the fetus, ultrasound challenges women’s privileged access to their own bodily interiors.

However, by contrasting her partner’s expectations about ultrasound with her own experience, Interviewee 3 is able to reposition herself as having privileged access to the meaning of her pregnancy. Through this process, she also contests anti-abortion framings of ultrasound visualization as the ultimate “truth” of fetal personhood. Although she invokes this framing, she also subverts it by constructing the scan as a process that lends moral authority to her decision to *end* her pregnancy. Moreover, she portrays her prior knowledge about this decision as inseparable from her encounter with fetal images: “I went into the scan hoping there was no heartbeat” and “it just confirms what you already know.”

A similar account was provided by another participant who asked spontaneously to look at a picture of her scan during her pre-abortion assessment:

I didn’t watch any of it. . . . But then afterwards we went into another room to talk about it and I actually asked to see the picture. And it got me on edge thinking if I wanted to go through with it or not. But *I took everything into the picture* and I still decided to go through with it.

Siân: Do you know why it was that you wanted to have a look at the picture, or?

No. It was just like a little bit of me that in a way wanted to look at it to be like, “Sorry,” and a bit to make sure that I did actually want to get rid of it. (Interview 4 [emphasis added])

This participant had previously described the arguments that her pregnancy had generated with her partner, which led her to decide that it could not continue. As well as portraying ultrasound as a means to test and reinforce this decision (taking “everything into the picture”), she also offers another depiction of the significance of visual encounters with the fetus. Specifically, she describes wanting to acknowledge and apologize to her fetus prior to abortion. Similar rationales for viewing are also reported in existing studies that have explored women’s responses to pre-abortion ultrasound (Graham, Ankrett, and Killick 2010; Kimport et al. 2012; Wiebe and Adams 2009). However, this literature does not consider the way in which women’s accounts simultaneously invoke and subvert maternal-fetal bonding discourse by constructing looking at the fetus as a process of ending (rather than beginning) a relationship.

Notably, women interviewed for this study often argued that social norms surrounding ultrasound make it difficult to use this technology as a means of acknowledging/remembering the fetus:

She asked if I wanted to see it, and after you’ve been through it and you’ve got nothing left of what was for me my first child it’s just like I kind of wish I’d said, “Yeah, I want to see it,” or “Yeah, I want to know more about it.” But I didn’t get to. . . . She said, “We don’t normally show the screens, but do you want to?” And obviously I didn’t want my boyfriend to hear me say, “Oh yes, I want to see it.” Because that might have upset him as well so I was just like, “No, I’d rather not know.” But now I’d kind of rather I did know. (Interview 10)

Similar expectations were described in a survey-based study of pre-abortion care in England (Graham, Ankrett, and Killick 2010), which found that many women wanted to view their ultrasound, but felt unable to ask.

## A Technology of Medical Objectification

In different ways, the accounts considered in the previous section both draw upon and “situate” characterizations of ultrasound as a technology that involves emotionally significant visual encounters between pregnant women and their fetuses. In this section, I use three examples to explore an alternative interpretative repertoire through which participants constructed the experience of having an ultrasound prior to abortion. I illustrate the ways in which participants positioned ultrasound as part of an objectifying medical gaze that produces knowledge about pregnant and fetal bodies. Through this repertoire of “medical objectification,” I suggest, ultrasound becomes disconnected from the production of maternal-fetal relationships and can even be mobilized as a tool of fetal *de-personification*.

### Participating in a Medical Assessment of the Pregnant Body

Several participants suggested that because of the way in which ultrasound is practiced within abortion clinics, being scanned was a relatively unremarkable method of obtaining useful medical knowledge about their bodies. For example, one woman presented ultrasound as part of a series of medical assessments that helped to facilitate the ending of her pregnancy:

Basically they just checked my details, went through my reasoning for why I was having the abortion, and then they said they’d do a blood test and a scan just to date the pregnancy, which they just did like a pinprick on my finger, and checked my iron levels. And then I had the dating scan and then they booked the appointment at [*clinic*], like, straightaway . . .

Siân: And you said as part of that you had a dating scan. Was that something that you sort of knew was going to happen or expected?

Yeah, they, the doctor, did tell me they would do that.

Siân: And how did you find—the reason I’m asking is that we don’t know much about what it’s like for women to have a scan—if it’s alright, or?

She basically just told me to lay down and that she would push quite hard just sort of above my bladder area and that she would turn the screen away and turn the sound off so I didn’t hear or see anything. So it literally was just like somebody rolling a ball over the, over your tummy, sort of your lower tummy area. (Interview 24)

As with several of the accounts considered previously, this participant foregrounds her bodily involvement during the scan. However, while other participants portrayed their embodied experiences of ultrasound as intrinsically connected to visual representations of maternal-fetal bonding in popular culture, Interviewee 24 offers a different characterization. She suggests that without a viewing screen (or sound), ultrasound is simply “a ball rolling over your tummy,” that is, the clinical probing of a pregnant woman’s abdomen.

### Engaging in Situated Scientific Spectatorship

Another participant, who chose to watch the viewing monitor, also offered an account of ultrasound that contrasts with those considered in the previous section. She described the act of viewing the interior of her pregnancy as a diagnostic process that rendered the fetus as a separate and alien presence *within* her own body. Far from personifying the fetus, her account of her experience of ultrasound locates the fetus as an invading, and removable, biological object:

I had to have a scan and she asked me if I wanted to see what she could see? She asked me how much info I wanted. And I was really interested at the time so I wanted to have a look and she was happy to let me. And she explained a lot about the sort of biology, which I was really interested in as well. . . . I think it’s quite a funny one because I think in sort of films and stuff the first scan is portrayed as if, you know, beautiful, really emotional, first time you see the baby, etc. A really kind of pivotal moment. And actually it wasn’t ever like that in my experience. And I was just kind of interested about the science. I was like, goodness, that’s what’s inside me. How bizarre! And it wasn’t this gorgeous fluffy turning point. It was—and I do, I have wondered since if/when I do have children and I do have a scan again how differently I’ll feel. Because I’m sure, well, if I do have children I’m sure I’ll be emotionally involved [and, you know, rooting for the process to work], etc. So I wonder if the scan will be a different process altogether. But for me at that time it was just complete science. It was just almost like when the dentist takes a tooth out and says, “Do you want to take your tooth home with you?” you know, it was just science and intrigue. And there was no emotional attachment. There was no sort of, “Oh gosh, now that I see what it is have I made the wrong decision?” None of that at all. (Interview 20)

Although this participant describes her experience of ultrasound using an interpretative repertoire of medical objectification, her account also locates medical objectification as a socially contingent process. Significantly, she portrays the construction of a “medical” vision of her pregnancy as dependent upon her interactions with the nurse, who narrated the scan using biological discourse. She also emphasizes the social context in which she found “the science” fascinating, connecting this to her feelings about her pregnancy at that time. Earlier in the interview she had described how, on finding out she was pregnant, she knew instantly that she did not want to be: Pregnancy was completely unsustainable and undesired at that time in her life. Correspondingly, she noted that if the circumstances were different and she wanted to be pregnant, then she would expect ultrasound to be a very different experience.

### Being “Early”: Medical Objectification as Moral Resource

Interviewee 20 (above) suggests that the objectifying possibilities of ultrasound are dependent upon the social context of the particular pregnancy in which one is invited to participate in a “scientific” account of its meaning. However, other participants mobilized this repertoire very differently, suggesting that the medical knowledge produced by ultrasound can *determine the meaning of being pregnant*:

They couldn’t do the ultrasound on the stomach. . . . They had to do it through the vagina because it was [literally] they couldn’t pick up anything. And so she confirmed it as six weeks and five days. . . . It was better than a Pap smear or anything, really. It was just—that was fine. Like, I didn’t want to look at the thing that she was—look at the monitor. But not a big deal, I just sort of, yeah, it really was fine. And then maybe even doing that made me feel like, you know, if I was having an ultrasound on my stomach, I don’t know, just from movies and everything, I might have felt like it was—I sort of almost preferred that it was that way because it didn’t feel as, um, as what you’d expect an ultrasound to be like for pregnancy. Do you know what I mean? Like, I just sort of felt like if it was on my stomach I’d feel like I was, like, someone off TV getting a . . . you know. It would have felt a bit too . . . I don’t know, it just, it did make me feel better but also that, you know, it was so small and so new that it couldn’t even come up like that. Like, I think definitely the period of time did seem to have an impact on me. . . . I wonder whether I would have . . . any longer I would have felt worse. But because it was so early and that, yeah, I sort of, I did feel better that it wasn’t. You know, the people outside had those things, like, showing what eight or nine weeks or something looked like, and if I’d been on the dot of what the posters there were showing or, you know what I mean, [that might] actually. But I knew that it wasn’t that far progressed. (Interview 23)

Echoing many elements of the accounts considered previously, this participant suggests that simply “not looking” is insufficient to disrupt the normative sociotechnical script of ultrasound. She links particular sets of practices to expectations about what ultrasound “should” be like in pregnancy, and highlights the way these expectations are entrenched in the visual culture of film and television. However, she also sets up a series of contrasts between these expectations and her own experience. Conducted vaginally, rather than via the abdomen, ultrasound becomes a different kind of practice entirely. As well as disrupting an anticipated sensory experience, the vaginal scan objectifies the participant’s pregnancy as literally *invisible*: It is simply too “small and . . . new” to be seen and dated using conventional medical methods. In turn, this enables her to contest the anti-abortion framing of the meaning of her pregnancy that she describes encountering (in the form of protestors wielding posters depicting fetal development) outside of the clinic. Ultrasound offers “objective” evidence that her pregnancy does not resemble those that are the focus of anti-abortion protest.

However, this account also highlights the ambiguous resource provided by a repertoire of medical objectification. Un-situated deployments of this repertoire suggest that the meaning of pregnancy is defined by ultrasound measurements, as opposed to pregnant women’s knowledge of their embodied social circumstances. Constructed in this way, the same tool of medical dating that defines Interviewee 23’s pregnancy as developmentally “insignificant” has the capacity to position other women’s pregnancies very differently.

## Conclusion

Feminist theory has countered anti-abortion portrayals of ultrasound images as “objective” evidence of fetal personhood by deconstructing such depictions, offering alternative readings of these images, and emphasizing the plurality of ultrasound across different contexts of its use. This article has contributed to this project through an analysis of women’s accounts of a context of pregnant embodiment that has been neglected by existing feminist research concerning ultrasound. In this concluding discussion I summarize the ways in which my engagement with women’s accounts of ultrasound prior to abortion contributes to feminist understandings of this technology.

A key concern within feminist writing about ultrasound is that pregnant women are erased from ultrasound images (Petchesky 1987), facilitating “fetal-centred” (Steinberg 1991, 179) discussion of abortion. However, Roberts (2012b) illustrates that in some contexts, it is possible to generate alternative representations of ultrasound that foreground pregnant women’s bodily involvement (see also Palmer 2009a). This possibility is likewise highlighted by this study. As my analysis has illustrated, several participants described ultrasound as providing visual, or other, information about *their own bodies*. Others positioned their bodily involvement as central to the generation of ultrasound images. In particular, women talked about feeling the probe gel being placed on their abdomen, feeling the probe pushing into their abdomen, or discovering that a probe had to be inserted into their vagina in order to visualize their uterus.

The depiction of pregnant subjects as central to the practice of ultrasound was a cross-cutting feature of participants’ accounts and represents one key way in which the interview data could be said to destabilize dominant depictions of the technology. However, I have also argued that participants employed two distinct interpretative repertoires in describing their experiences, each of which poses other challenges to narrow accounts of ultrasound’s meaning. In the case of the repertoire of medical objectification, participants portrayed ultrasound as a tool of medical knowledge production de-coupled from processes of fetal personification. Drawing on this repertoire, participants described ultrasound as an opportunity to satisfy scientific curiosity about biological events taking place inside their bodies, as one of a series of medical assessments to which their own bodies needed to be subjected to enable their abortion, and as evidence that the embryo/fetus within their bodies was developmentally and morally insignificant. All such depictions represent a radical departure from the characterization of ultrasound as an experience of “maternal-fetal bonding.”

These findings resonate with cross-cultural research concerning prenatal ultrasound, which highlights that this technology is not always linked to the personification of the fetus. Rather, in many contexts, ultrasound is primarily a medical tool used to objectify maternal/fetal bodies and generate knowledge about the “viability” (and potential nonviability) of a pregnancy (Gammeltoft 2007; Mitchell and Georges 1997; Morgan 2000). However, this article also extends existing studies by illustrating that in the context of abortion care, the repertoire of ultrasound as medical objectification can become *actively* enrolled in projects of fetal de-personification.

At the same time, I have suggested that unsituated uses of the repertoire of medical objectification provide an ambiguous resource to women who decide to end their pregnancies. Women can deploy ultrasound’s objectification of the fetus as evidence of its developmental—and moral—insignificance, if the technology locates their pregnancy as sufficiently “early.” Simultaneously, however, this constructs the meaning of pregnancy as defined by medical measurements of fetal development, as opposed to pregnant women’s knowledge of their embodied social circumstances. This assumption is central to the regulation of abortion in England, Scotland, and Wales, and has underpinned attempts to restrict women’s access to abortion at later gestations of pregnancy (Palmer 2009b; Science and Technology Subgroup 1991; Sheldon 1997).

The other interpretative repertoire that participants drew on to characterize experiences of ultrasound contrasts strikingly with the repertoire of medical objectification. Using a repertoire of “situated visual relationships,” participants depicted ultrasound as a site at which emotionally significant, yet socially specific, visual encounters between pregnant women and their fetuses take place. In doing so, they challenged the “conflation of seeing with knowing” (Palmer 2009b, 174) that is central to anti-abortion rhetoric, and offered representations of ultrasound as something other than an experience of maternal-fetal bonding. For example, some women suggested that ultrasound evokes visualizations of joyful maternity but rejected this sociotechnical script as incompatible with their own feelings about their pregnancies. Others described engaging in emotionally significant visual encounters with their fetuses prior to abortion, but subverted anti-abortion depictions of the meaning of this process. They argued that their decisions to end their pregnancies were reinforced through witnessing the “reality” of their fetuses, and also emphasized the way in which this reality was constructed through their own prior knowledge about the meaning of their pregnancies. Similarly, other participants suggested that when the decision has been made to end a pregnancy, ultrasound can represent a means of articulating the loss, or ending, of a relationship—rather than signifying its beginning.

However, while participants did challenge depictions of ultrasound as an experience of joyful maternal spectatorship, their use of the repertoire of situated visual relationships was nonetheless oriented to this dominant portrayal of ultrasound’s meaning. This orientation was likewise visible when women drew on a repertoire of medical objectification, which was often used to construct contrasts with popular portrayals of ultrasound. The difficulties involved in generating alternative accounts of this sociotechnical practice were stated explicitly by several participants. For example, women who chose not to look at the viewing screen drew attention to the lack of representation of their experiences within visual culture. They argued that in film and television, ultrasound is depicted as a moment of joyful maternal-fetal bonding, excluding the possibility that this technology, as well as women’s pregnancies, might hold very different meanings. Relatedly, other participants highlighted the difficulties involved in pursuing visual encounters with the fetus that transgress normative expectations about the basis of these encounters (i.e., that they might involve acknowledging the loss of a relationship rather than joyfully anticipating its development). Collectively, their accounts highlight the importance of changing the stories about ultrasound and pregnancy that “get to travel” (Taylor 2008, 14), and suggest that one way feminist research could facilitate this process would be to expand empirical analyses of this technology to encompass its use during abortion provision.

## Notes

1. Any attempt to reify dichotomous “categories” of pregnancy is problematic, because (as participants in my study attest) the meaning of pregnancy can alter over time. In this article, I construct a pragmatic distinction between “pregnancies that women plan to carry to term” and “pregnancies that women decide to terminate,” while acknowledging that the former sometimes become the latter, and vice versa. I use these descriptive terms in order to avoid characterizing pregnancies in more normative terms as either “wanted” or “unwanted’’ (although I do use this language in situations when it is employed by participants themselves). Research indicates that the terms *wanted*/*unwanted* are often regarded as problematic by women who terminate their pregnancies: Pregnancies may be “wanted” but simultaneously impossible (Barrett and Wellings 2002). For this reason, I have also avoided characterizations of women as “willingly”/“unwillingly” pregnant (as used by Hopkins, Zeedyk, and Raitt [2005] in their analysis of antiabortion discourse): A woman may be “willingly” pregnant, but unable to continue with her pregnancy.

2. The proportions vary between studies.
